# Molecular analyses of H3N2 canine influenza viruses isolated from Korea during 2013–2014

**DOI:** 10.1007/s11262-015-1274-x

**Published:** 2016-01-25

**Authors:** EunJung Lee, Eun-Ju Kim, Bo-Hye Kim, Jae-Young Song, In-Soo Cho, Yeun-Kyung Shin

**Affiliations:** Viral Disease Division, Animal and Plant Quarantine Agency, 175 Anayngro, Anyang, Gyeonggido 430-757 Republic of Korea; Veterinary Drugs and Biologics Division, Animal and Plant Quarantine Agency, Anyang, Gyeonggido Republic of Korea

**Keywords:** H3N2 CIV, Canine influenza virus, Phylogenetic analysis, Molecular analysis, Korea

## Abstract

Canine influenza A virus (CIV) causes a respiratory disease among dog populations and is prevalent in North America and Asia. Recently, Asian H3N2 CIV infection has been of particular concern, with recent reports related to reassortants with pandemic 2009 strains, direct transmission from a human H3N2, a possibility of H3N2 CIV transmission to other mammals, and even the first outbreak of H3N2 CIVs in North America in April 2015. However, despite these global concerns, our understanding of how influenza A virus transmission impacts the overall populations of H3N2 CIVs remains incomplete. Hence, we investigated the evolutionary history of the most recent two Korean CIV isolates, A/canine/Korea/BD-1/2013 and A/canine/Korea/DG1/2014, along with 57 worldwide CIVs, using comprehensive molecular analyses based on genomic genotyping. This study presents that the new Korean CIV isolates are closely related to the predominantly circulating H3N2 CIVs with genotypes *K, G, E, 3B, F, 2D, F,* and *1E*, carrying several mutations in antigenic and host determinant sites. Also, our findings show that the genome-wide genetic variations within the H3N2 CIVs are low; however, two antigenic protein (HA and NA) analysis demonstrates genetic diversification of the H3N2 CIVs, which evolves independently between Korea and China.

**Electronic supplementary material:**

The online version of this article (doi:10.1007/s11262-015-1274-x) contains supplementary material, which is available to authorized users.

## Introduction

Influenza A virus (IAV) is highly contagious and causes respiratory diseases with symptoms ranging from minor to severe, depending on the host and the subtype of IAV [e.g., H5N1 and H7N7 as highly pathogenic avian influenza (HPAI)] [1]. It has infected various species, such as humans, other mammals (e.g., swine, equines, canines, and felines), and a broad range of domestic or wild birds [1, 2]. Like a typical RNA virus, it shows high mutation rates caused by poor proofreading during gene replication [3].Occasionally, mixed infections of IAV from different hosts can produce genetic recombination due to the eight-segmented genes, thereby generating a novel virus with new genomic genotypes that can cross host barriers [1, 2, 4]. Because of these characteristics, previous reassortants, known as the Spanish flu H1N1 (1918), the Asian flu H2N2 (1957), the Hong Kong flu H3N2 (1968), and the swine-origin pandemic H1N1 (2009), resulted in hundreds of thousands casualties and significant economic loss [1, 2, 4]. Accordingly, monitoring interspecies transmission events has drawn greater attention to preventing newly emerging IAVs with pandemic potential. Among the various mammalian hosts, dogs have been considered to have zoonotic capacity as an intermediate host because they are in close contact with humans as companion animals [1, 5]. More importantly, because the respiratory tract of the dog was found to have both α-2,6- and α-2,3-sialic acid-linked influenza virus receptors that are targets for influenza virus infection [6], the emergence of CIVs is expected to have a greater likelihood of interspecies transmission.

Since the first report of two different types of canine influenza virus (CIV)—H3N2 in the Republic of Korea as early as 2005 [7] and a major outbreak of H3N8 in the USA in 2004 [8]—dogs have played a key role in facilitating mammalian host adaptation of CIV and its subsequent spread. In light of phylogenetic analysis, the CIVs have formed two distinct lineage groups within the IAVs over the last decade—an Asian H3N2 avian-origin lineage and a North American H3N8 equine-origin lineage– as a result of a direct interspecies transmission from different hosts [8–11]. For this reason, the H3N2 CIVs have shown low levels of genetic diversity in the distinct lineage group belonging to the Eurasian avian lineages [10–20]; similarly, H3N8 CIVs have formed a single monophyletic group separated from the H3N8 equine lineage [8, 9, 21].

Until earlier this year, the geographic distribution of H3N2 CIV had been limited to Asian regions, such as the Republic of Korea, China, and Thailand [10, 12–17, 19, 20]; however, since the first outbreak in dogs in the USA in April 2015, it has rapidly spread to other Midwestern location from Chicago [22, 23]. In contrast, H3N8 CIV has undergone a discontinuous distribution in three disjunctive continental areas: the USA [21] since 2004, Australia in 2007 [24], and the United Kingdom in 2002 [25]. Although the long-distance transmission of CIVs has occurred across continents and country boundaries in recent years, the transmission mechanism and reassortment events through space and time remain unclear.

Further, the pandemic 2009 H1N1 and human H3N2 reassortants have occasionally jumped the host barrier, causing H3N2 CIV-pandemic mixed strains or complete transmission strains from human H3N2 [26–30]. The concern has been emphasized more because recent clinical studies of the transmission of H3N2 CIV have shown the possibility of infecting a broad host range, including dogs, cats, mice, ferrets, and guinea pigs [10, 17, 20, 31–35]. Accordingly, despite the limited sampling of H3N2 CIVs across geographic regions and over time, the occurrence of relatively many subtypes of IAV infection in dogs in Asia has resulted in a warning of a potential pandemic threat, exemplified by H5N1 (A/dog/Thailand-Suphanburi/KU-08/04) [36], H5N2 (A/dog/Shandong/JT01/2009) [37],H1N1 (A/canine/Beijing/cau2/2009) [30], and H9N2 (A/canine/Guangxi/1/2011) [38].

To date, phylogenetic studies and genetic analyses of H3N2 CIV have been broadly conducted [10–20]; however, genome-wide genotyping studies have been lacking. To fill this gap, we quantified the genotypes of CIV prevalent into Asia using currently available H3N2 CIV sequences with two recent South Korean isolates, and we then examined the patterns of genome-wide genetic variation of H3N2 CIVs.

This study was performed with the following specific objectives: (1) to reveal the evolutionary history of two recent Korean H3N2 CIVs, and (2) to investigate the global distribution and prevalence of the extant H3N2 CIV genotypes using various molecular analyses.

## Materials and methods

### Korean CIV isolates and RNA extraction

In this study, two Korean CIVs were collected from nasal swabs of pet dogs at animal hospitals in Seongnam in 2013 (A/canine/Korea/BD-1/2013) and in Daegu in 2014 (A/canine/Korea/DG1/2014), respectively. For the virus isolation, the IAVs were propagated in 10-day-old embryonated specific pathogen-free (SPF) chicken eggs (VALO, USA), and 3 days later, the allantoic fluid was harvested. The procedure was conducted according to the instructions from World Organization for Animal Health (OIE) terrestrial manual [39] and viruses from the first passage were used for RNA extraction. The viral RNAs in the allantoic fluid were extracted using the RNeasy Mini Kit (QIAGEN, USA), according to the manufacturer’s protocol. All of the influenza viral RNAs were confirmed using M segment primers previously described by Shin et al. [40] for the detection of IAVs.

### RT-PCR, cloning, and sequencing of the two Korean CIV isolates

To amplify the eight viral segments of the two Korean CIV isolates, universal primers from Hoffmann et al. (2001) [41] were used with modifications (primer sequences available on request). For the reverse transcription and PCR amplification reactions for sequencing, a One-Step RT-PCR kit (QIAGEN, USA) was used. The amplifications were performed in a total reaction volume of 20 µL, containing 5X RT-Buffer, 1 µL of dNTP mix (10 mM each), 0.5 µL of the RT-PCR enzyme mix, 2.5–3 µL of RNA template, and 3 µL of the primer sets (10 pmol/µL), according to the manufacturer’s instructions. The thermocycling conditions included 30 min at 50 °C for a reverse transcription step, 15 min at 95 °C as a heating step, and 30 cycles of PCR: 94 °C for 30 s, 55–58 °C for 30 s, and 72 °C for 1 min, followed by a terminal 10 min extension at 72 °C. All of the PCR reactions were performed in a BiometraT3000 thermal cycler (Biometra, the Netherlands) and then were analyzed on 1–1.5 % SeaKem LE agarose gel (Lonza, USA) containing RedSafe™ Nucleic Acid Staining Solution (iNtRon Biotechnology, South Korea), in 0.5X Tris–acetate-EDTA (TAE) buffer (BIOSESANG Inc., South Korea). The RT-PCR products were ligated to pGEM-T Easy Vectors with T4 ligase (Promega, USA), according to the manufacturer's instructions. After transformation of the ligated products into DH5α chemically competent E. coli (Enzynomics, South Korea), the transformed bacteria were left for 1 hour in S.O.C. medium (Invitrogen, USA) at 37 °C for recovery, and later, 200 µL of the transformation culture were plated onto an LB agar plate containing ampicillin (AMRESCO Inc., USA) (100 mg/mL), IPTG (Bioneer Corp., South Korea) (1 M) and X-gal (iNtRon Biotechnology, Korea) (20 mg/mL), which was cultured overnight at 37 °C. The obtained white colonies were screened by colony PCR, and the recombinant plasmids were isolated from the colonies with an IncloneTM Plasmid mini prep kit, version 2.0 (Inclone Biotech, South Korea), according to the manual. Each plasmid was sent to a commercial company (Macrogen, South Korea) for confirmation and characterization by sequencing. All of the sequences were edited using CLC Main Workbench software, version 6.9 (CLC Bio), and ambiguous bases were resolved by repeated sequencing. The BLAST search was used to determine the most similar sequences to those of the isolates. Based on the BLAST results, the two isolates were confirmed as H3N2 subtypes of CIV, showing particularly high homology with H3N2 Korean CIVs. The genomic sequences of the isolates have been deposited in GenBank under the following accession numbers: KR154318-KR154325 for A/canine/Korea/BD-1/2013 and KR154326-KR154333 for A/canine/Korea/DG1/2014.

### Sequence alignment and composition

For genotyping and phylogenetic studies of H3N2 CIVs, all of the publicly available H3N2 CIV sequences over the past 8 years (from 2004 to 2012) as of February 2015 were downloaded from the NIAID IRD [42] (Influenza Research Database: http://www.fludb.org), with representatives of the different subtypes of CIVs (e.g., H3N1, H3N8, H5N1/N2, H1N1, H9N2) (Supporting Table S1). Each segment was aligned with two newly isolated H3N2 Korean CIVs, using MAFFT, version 7 [43], from http://mafft.cbrc.jp/alignment/server/, according to the FFT-NS-2 strategy with default parameters. Finally, the total of 59 CIV genomic sequences were used for this study, and their data composition was as follows: 5 countries: South Korea (16), China (39), Thailand (2), the USA(1), and Australia (1); and each segment length (in most cases): PB2 (2277 bp), PB1 (2271 bp), PA (2148 bp), HA (1698 bp), NP (1494 bp), NA (1413 bp), M (979 bp), and NS (838 bp). The following segments with various sequences were used to estimate genetic variation and structure: PB2 (54), PB1 (53), PA (54), HA (54), NA (52), NP (55), M (57), and NS (55). However, for accurate molecular analyses, different genotype segments caused by direct transmission or reassortment events and strains with missing segments were manually excluded from 59 CIV genomic sequences, depending on the purpose of the analysis. Finally, 40 H3N2 CIVs with genomic sequences (i.e., the total number of characters: 13,118 bp) were used to reveal the evolutionary position of the two Korean CIVs among the H3N2 CIVs.

### Genomic genotyping

The genotype of each of eight segments from the 59 CIVs were determined by FluGenome (http://www.flugenome.org/) [44], which defines significant lineages classified by 10 % nucleotide difference based on p-distance (i.e., the proportion of sites that differ between two sequences). Using FluGenome, the default thresholds for the sequence comparison using the BLAST algorithm were set to at 95 % identity, 95 % coverage, 96 word-size, and 1e-5 E-value. The genotype nomenclature for each segment was referred to by the names previously determined by Lu et al. [44] and Dong et al. [45].

### Phylogenetic analyses

To infer phylogenetic relationships between the two Korean H3N2 CIV isolates and worldwide CIVs, the following phylogenetic methods were employed depending on different datasets: (a) RAxML 7.4.2 [46] implemented in raxmlGUI, version 1.3 [47] for a maximum likelihood (ML) method; (b) Garli 2.01 [48] using genetic algorithm approaches for the ML search, allowing all possible submodels of the GTR (General Time Reversible) model; and (c) MrBayes, version 3.3.2 [49], for Bayesian inference (BI) using the Markov chain-Monte Carlo (MCMC) method.

Before phylogenetic analyses, nucleotide substitution models and the best-fit partitioning schemes were estimated to consider the different evolutionary rates of each segment and evolutionary rate per site at codon by two programs: (a) jModelTest, version 2.1.6 [50] for nucleotide substitution models of a single partition (i.e., each segment and concatenated genome) under the Akaike Information Criterion (AIC), corrected AIC (AICc), and Bayesian Information Criterion (BIC); and (b) PartitionFinder, version 1.1.1 [51], for the best partitioning schemes according to the BIC. Because M and NS segments produce two polyproteins each (i.e., M1, M2, NS1, NS2) due to ribosomal slippage during translation [2], codon partitions were not applied for them. The detailed information of parameter settings for each analytical method is further described in the supporting information (see S3).

Additionally, the genetic distance between and within genotype lineages in all of the trees was calculated using p-distance in MEGA, version 5.1 [52]. For the genetic distance, all of the ambiguous positions were removed for each sequence pair. Based on the genetic distance results, the most divergent group was used as an outgroup for each segment tree result.

### Genetic variation of H3N2 CIVs

The genetic diversity and population structure of each segment were estimated using DnaSP, version 5.1 [55], and PopART, version 1.7 [56]. The following summary statistics were calculated: polymorphic (segregating) sites (S); parsimony informative sites (Pi); the number of haplotypes (i.e., the set of DNA variations) (h); haplotype diversity (Hd); nucleotide diversity (π); average number of nucleotide differences (k); and Tajima’s D (i.e., to estimate demographic events). In addition, to estimate genealogical relationships of haplotypes for HA and NA, their haplotypes were further analyzed using a median-joining network [57]. Analysis of molecular variance **(**AMOVA), implemented in PopART, was used to estimate the correlation of haplotypic diversity at different levels of subdivision: South Korea and China including Thailand. Subsequently, the deduced amino acid sequences of eight segments from the two Korean CIVs were compared with those of other H3N2 CIVs based on several functional sites (e.g., cleavage sites, glycosylation sites, receptor-binding sites, and antigenic sites), along with H3N2 CIV-specific host markers, which have been described in recent studies [16, 19, 20, 11]. Also, for the comparison with other IAVs, amino acids with a high frequency of occurrence at each site of H3N2 IAV genomic sequences from avian, swine, and human hosts were further investigated using the Analyze Sequence Variation (SNP) tool in IRD.

## Results

### CIV genotyping

In the genotype profiles of 59 CIVs by FluGenome, the conserved genotypic patterns of H3N2 CIVs were identified (Table 1). Nine distinct genotypes were observed in CIVs from five countries during the last decade (2004-2014). However, most of them were abruptly generated by reassortment events or by transmission. Two genotypes were commonly distributed into the two continents: Asia (*K, G, E, 3B, F, 2D, F,* and *1E*) and North America (USA) (*C, I, G, 3F, C, 8B, E, and 1D*). Within the Asian region (Korea, China, and Thailand), the genotype frequency was more than 73 % (42/57). The two newly isolated CIV isolates from Korea also possessed the same genotype combinations detected in Asian CIVs. Interestingly, it was in Korea that four CIV reassortants with a pandemic 2009 strain were observed (Table 1). In contrast, five different subtypes with different genotype patterns of CIVs were observed in China and Thailand, along with equine-origin H3N8 in the USA and Australia (Table 1). Notably, the PB1, NP, M, and NS segments possessed each persistent genotype—G, F, F, and 1E, respectively—despite several reassortment and transmission events. In particular, the F genotype of the M segment was indicated as the most common among CIVs in Asia.

**Table 1:** Genotype profiles of H3N2 CIVs and different subtypes of CIVs

Country	CIV isolates^a^	Year^b^	Segment Genotype^c^								Events^d^	Reference^e^
			PB2	PB1	PA	HA	NP	NA	M	NS		
Korea	Korea/01/2007	2007	***K***	***G***	***E***	***3B***	***F***	***2D***	***F***	***1E***		[62]
	Korea/GCVP01/2007	2007	–	–	–	–	–	–	–	–		[10]
	Korea/LBM412/2008	2008	–	–	–	–	–	–	–	–		[63]
	Korea/SNU9046/2009	2009	na	na	na	na	na	na	na	–		[64]
	Korea/CY001/2010	2010	na	na	na	–	na	–	–	na		[31]
	Korea/CY005/2010	2010	na	na	na	–	na	–	–	na		
	Korea/CY009/2010	2010	–	–	–	–	–	–	–	–		
	Korea/1/2010 (H3N1)^‡†^	2010	*C*	*D*	–	–	*A*	*1F*	–	*1A*	R1	[26]
	Korea/KRIBB01/2011	2011	–	–	–	–	–	–	–	–		Unpublished
	Korea/MV1/2012	2012	–	–	–	–	–	–	–	–		[65]
	Korea/S1/2012	2012	–	–	–	–	–	–	–	–		Unpublished
	Korea/VC378/2012^†^	2012	–	–	–	–	–	–	–	*1A*	R2	Unpublished
	Korea/VC123578/2012^†^	2012	*C*	*D*	–	–	*A*	–	–	*1A*	R3	Unpublished
	Korea/VC125678/2012 (H3N1)^‡†^	2012	*C*	*D*	–	–	*A*	*1F*	–	*1A*	R4	Unpublished
	Korea/BD-1/2013*	2013	–	–	–	–	–	–	–	–		This study
	Korea/DG1/2014*	2014	–	–	–	–	–	–	–	–		This study
China	Guangdong/1/2006	2006	–	–	–	–	–	–	–	–		[15]
	Guangdong/2/2006	2006	–	–	–	–	–	–	–	–		
	Guangdong/1/2007	2007	–	–	–	–	–	–	–	–		
	Guangdong/2/2007	2007	–	–	–	–	–	–	–	–		
	Guangdong/1/2011	2011	–	–	–	–	–	–	–	–		[66]
	Guangdong/05/2011	2011	–	–	–	–	–	–	–	–		
	Guangdong/2/2011	2011	–	–	–	–	–	–	–	–		[67]
	Guangdong/3/2011	2011	–	–	–	–	–	–	–	–		[12]
	Guangdong/04/2011	2011	–	–	–	–	–	–	–	–		
	Guangdong/12/2012	2012	–	–	–	–	–	–	–	–		[68]
	Guangdong/23/2012	2012	–	–	–	–	–	–	–	–		Unpublished
	Beijing/cau2/2009 (H1N1)^‡†^	2009	*C*	*D*	–	*1A*	*A*	*1F*	–	*1A*	P	[30]
	Beijing/253/2009	2009	–	–	–	–	–	–	–	–		[16]
	Beijing/295/2009	2009	–	–	–	–	–	–	–	–		
	Beijing/305/2009	2009	–	–	–	–	–	–	–	–		
	Beijing/359/2009	2009	–	–	–	–	–	–	–	–		
	Beijing/362/2009	2009	–	–	–	–	–	–	–	–		
	Beijing/364/2009	2009	–	–	–	–	–	–	–	–		
	Beijing/418/2010	2010	–	–	–	–	–	–	–	–		
	Beijing/420/2010	2010	–	–	–	–	–	–	–	–		
	Beijing/511/2010	2010	–	–	–	–	–	–	–	–		
	Beijing/1028/2010	2010	–	–	–	–	–	–	–	–		
	Liaoning/1578/2010	2010	–	–	–	–	–	–	–	–		
	Liaoning/1585/2010	2010	–	–	–	–	–	–	–	–		
	Liaoning/27/2012	2012	–	–	–	–	–	–	–	–		[69]
	Liaoning/H6/2012	2012	–	–	–	–	–	–	–	–		Unpublished
	Jiangsu/01/2009	2009	–	–	–	–	–	–	–	–		[20]
	Jiangsu/02/2010	2010	–	–	–	–	–	–	–	–		
	Jiangsu/03/2010	2010	–	–	–	–	–	–	–	–		
	Jiangsu/04/2010	2010	–	–	–	–	–	–	–	–		
	Jiangsu/05/2010	2010	–	–	–	–	–	–	–	–		
	Jiangsu/06/2010	2010	–	–	–	–	–	–	–	–		
	Zhejiang/1/2010	2010	–	–	–	–	–	–	–	–		[14]
	Nanjing/11/2012	2012	na	na	na	–	–	–	na	na		Unpublished
	Heilongjiang/L1/2013	2013	–	–	–	–	–	–	–	–		Unpublished
	Guangxi/1/2011 (H9N2)^‡†^	2011	*G*	–	–	*9C*	–	*2B*	–	–	T1	[38]
	Guangxi/L1/2013^†^	2013	*A*	*D*	*B*	*3A*	*A*	*2A*	*B*	*1A*	T2	[18]
	Guangxi/L2/2013^†^	2013	*A*	*D*	*B*	*3A*	*A*	*2A*	*B*	*1A*	T3	
	Shandong/JT01/2009 (H5N2)^‡†^	2009	–	–	*D*	*5* *J*	–	2F	–	–	T4	[37]
Thailand	Thailand/CU-DC5299/2012	2012	–	–	–	–	–	–	–	–		[19]
	Thailand-Suphanburi/KU-08/04 (H5N1)^‡†^	2004	–	–	*D*	*5* *J*	–	1 J	–	–	T5	[36]
USA	Florida/43/2004 (H3N8)^‡†^	2004	*C*	*I*	*G*	*3F*	*C*	*8B*	*E*	*1D*	T6	[8]
Australia	Sydney/6085/2007(H3N8)^‡†^	2007	na	na	na	*3F*	na	*8B*	*E*	na		[24]

^a^H3N2 CIV isolates: Bold with an asterisk (*) denotes the newly isolated Korean CIV isolates; ^‡^ Different subtypes CIVs; ^†^ Transmission or reassortment

^b^Isolation year of the CIV isolates

^c^Genotypes from PB2 to NS: the most common genotype found in Asia is described in bold and italics on the top: (PB2)-K; (PB1)-G; (PA)-E; (HA)-3B; (NP)-F; (NA)-2D; (M)-F; (NS)-1E. There are no sequences available for the analysis: ‘na.’ Each hyphen represents the same common genotypes, and different genotypes are presented as the corresponding genotype with italics

^d^Segment change events based on genotype profiles were described in Dong et al. [45]: **Reassortant (R)**: R1,3,4 with 2009 S-OIV H1H1; R2 NS reassortment; **Transmission (T)**: T1 from domestic poultry H9N2 (G, G, E, 9C, F, 2B, F, 1E); T2-3 from classic human H3N2 (A, D, B, 3A, A, 2A, B, 1A); T4 from domestic poultry H5N2 (K, G, D, 5 J, F, 2F, F, 1E); T5 from domestic poultry H5N1 (K, G, D, 5 J, F, 1 J, F, 1E); T6 from equine H3N8 (C, I, G, 3F, C, 8B, E, 1D); **Pandemic (P)**: 2009 S-OIV H1N1 (C, D, E, 1A, A, 1F, F, 1A)

^e^Reference lists relevant to CIVs

### Phylogenetic analyses based on genotyping

**Fig. 1:**
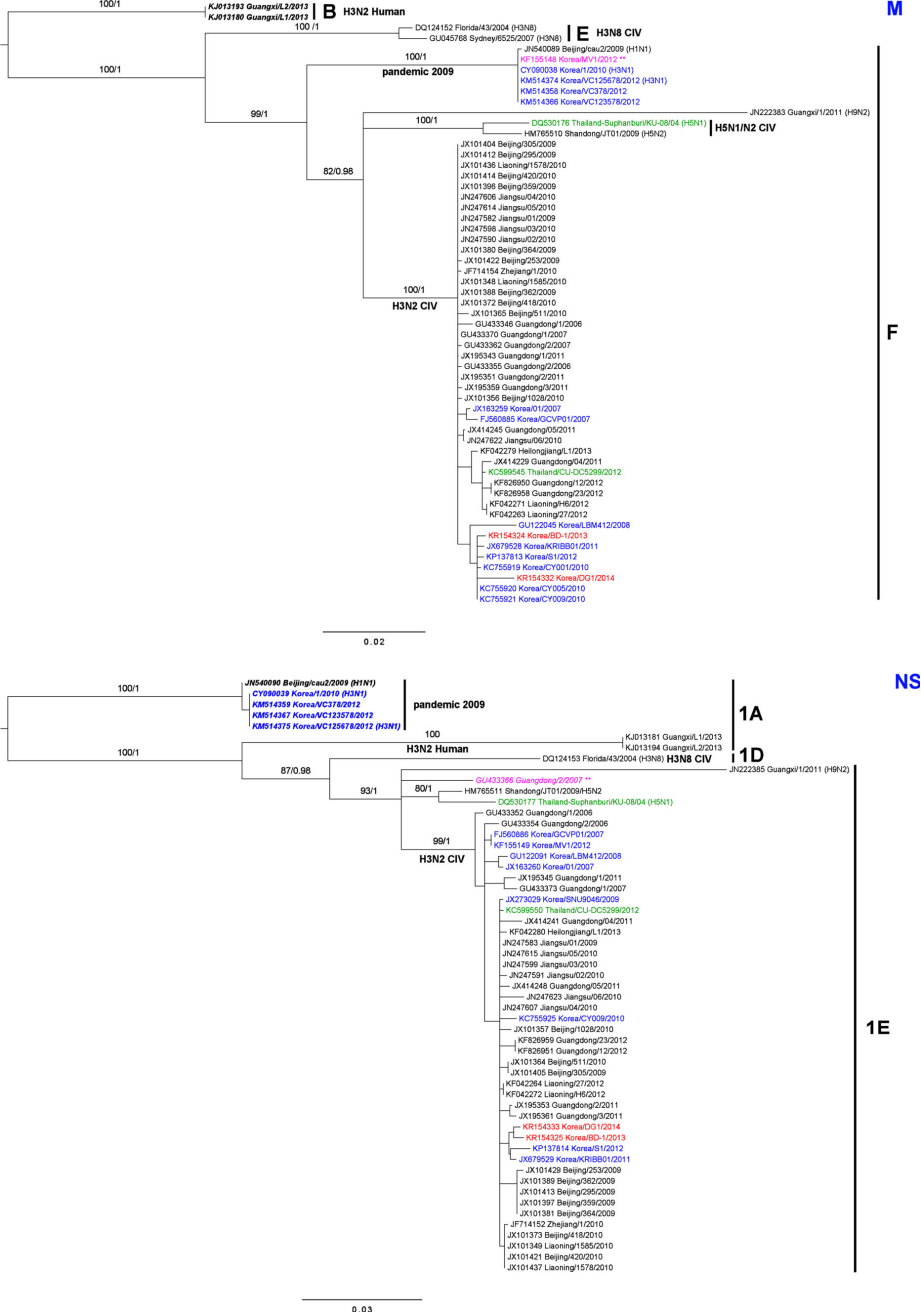
Phylogenetic trees of CIVs according to genotypes based on M (**a**) and NS (**b**). Korean isolates are shown in *blue*; CIVs isolated in the present study are shown in *red* and the Thailand isolate in *green*. The remaining isolates are from China. Numbers at nodes indicate support values for maximum likelihood (RAxML) and posterior probabilities for Bayesian inference (MrBayes). Only support values >50 are shown. H3N2 isolates belonging to distinct lineages separated from H3N2 CIVs are in magenta with *two asterisks*. Genotypes for each group are indicated with *vertical line*s. Each tree is rooted on the most distant group or isolate, based on p-genetic distance (See Table S2)

The phylogenetic relationships between the 57 H3N2 CIVs and the two recent Korean CIV isolates are presented in S1 Fig. and Fig. 1. As selected results, M and NS segments are presented in Fig. 1. Overall, the ML and BI trees showed the exactly same topologies, in which each segment was divided into several subgroups corresponding to genotype lineages. A few genotype lineages were not fully resolved in ML trees with less than 70 supporting values (e.g., 61 of C in PB2; 68 of 3B in HA3; see Supporting Fig. S1), compared to BI (i.e., > 0.87 posterior value). As shown in Fig. 1, each segment exhibited a monophyletic group of H3N2 CIVs within well-established intersubtypic CIVs from different hosts. All of the segments of Korean two isolates belonged to the H3N2 CIV lineage with the dominant genotypic pattern [i.e., (*K, G, E, 3B, F, 2D, F, 1E*)]. In particular, all eight of the segments of the two Korean CIVs were grouped together with the previous Korean CIVs isolated between 2010 and 2012. Moreover, the inferred phylogenetic trees from all of the segments exhibited distinct clades of variants separated from the H3N2 CIVs. The reassortants were clearly located outside the main H3N2 CIV clade, demonstrating that their segments are variants caused by reassortment events or direct transmission with different origins. Such differentiation was also observed in other segments according to the two events (S1 Fig.).

Along with the results, inconsistent phylogenetic relationships related to genotype lineages were discovered in internal segments. This finding reflected a historical reassortant origin of each genotype lineage. As an illustration, A/canine/Korea/VC123578/2012, which is a reassortant with a pandemic 2009 strain, was clustered in the same genotype (F) with the main H3N2 CIV in M segment, whereas it was separated from the main H3N2 CIV by the 1A genotype in the NS segment (Fig. 1). The results were plausible that a pandemic 2009 strain was composed of eight segments from four different origins [4]: avian (PB2, PA), swine (classical swine: HA, NP, NS and Eurasian avian-like swine: NA, M), and human (PB1). Thus, pandemic 2009 in the PB2, PA, or M segment showed close relationships with the main H3N2 CIV clade having an avian origin; otherwise, they were separated into different genotypes (Fig. 1 and Fig. S1). For a clear explanation of the topology patterns, the genetic distances between genotype lineage and different subtypes were calculated using p-distances, and they are summarized in Table 2 and Table S2, according to common group names. The closest group from the main H3N2 CIV in internal segments was H5N1, H5N2, or H9N2 at 4.7–10 %. In contrast, the most distant group from H3N2 CIV was mostly H3N2 human at 12.7–19.1 %. However, in PB1 and NS, the farthest genetic distances were H3N8 CIV at 18.7 % and pandemic 2009 at 16.4 %, respectively.

**Table 2:** Estimates of evolutionary divergence over sequence pairs between groups in M and NS segment

	H3N2 CIV	H5N1	H5N2	pandemic 2009	H3N8 CIV	H9N2	H3N2 human
H3N2 CIV		0.073	**0.064** ^**a**^	**0.164** ^**b**^	0.110	0.119	0.156
H5N1	0.080		0.028	0.174	0.111	0.116	0.147
H5N2	**0.073** ^**a**^	0.024		0.161	0.097	0.110	0.136
Pandemic 2009	0.089	0.089	0.088		0.163	0.209	0.186
H3N8 CIV	0.095	0.116	0.110	0.111		0.144	0.159
H9N2	0.113	0.117	0.117	0.127	0.149		0.167
H3N2 human	**0.127** ^**b**^	0.125	0.130	0.135	0.122	0.134	

Lower matrix: M segment; Upper matrix: NS segment

^a^Minimum divergence

^b^Maximum divergence from H3N2 CIVs is represented by bold numbers for each segment

**Fig. 2:**
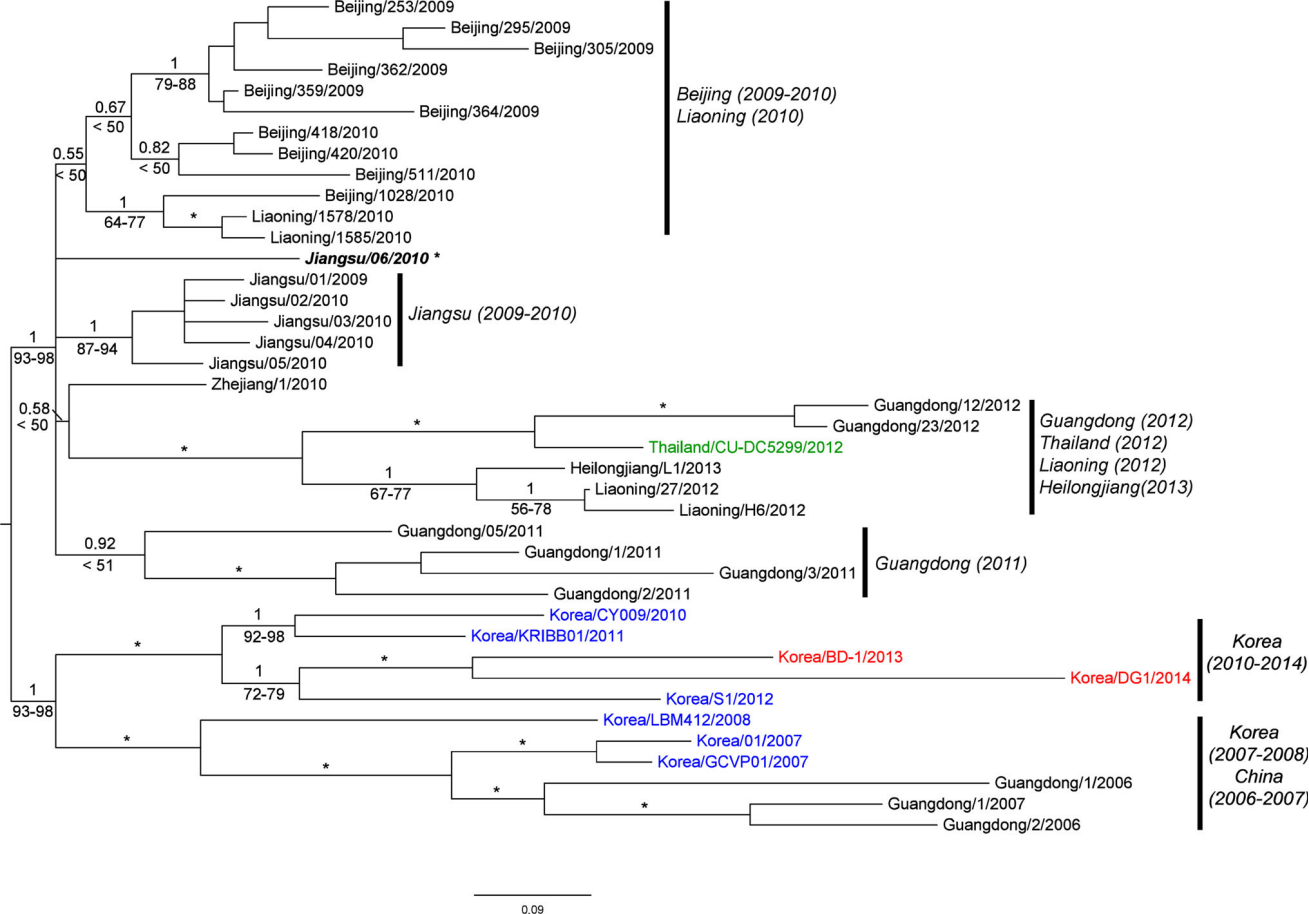
Bayesian inference phylogenetic tree of 40 H3N2 CIVs based on the concatenated eight segments. Korean isolates are shown in *blue*; CIVs isolated in the present study are in *red*, and the Thailand isolate is in *green*. The remaining isolates are from China. Support values from ML analyses are presented with posterior probabilities for Bayesian inference. Only support values >50 are shown. An *asterisk* at the node indicates high support values presenting more than 98 in ML and 1.0 of BI analyses in all of the analyses

A genome-wide phylogenetic tree, reconstructed under specific partition schemes (e.g., by codon and by segment) suggested by Partition Finder, produced similar topologies, except for the differences in support values in all of the analyses (Fig. 2). As mentioned above, all of tree results supported that the two Korean CIVs isolated in this study were closely related to the most recent Korean isolates. Those results were consistent with individual segment analysis. The phylogenetic results of their full genome showed that A/canine/Korea/S1/2012 was the closest isolate.

### Genetic variation of each segment

Genetic analysis of each segment from H3N2 CIVs was conducted using different numbers of sequences due to incomplete sequencing, and the results were described in Table 3. Overall, various genetic parameters did not show notable differences among segments, but HA, NA, and NP are more genetically diverse than the other segments. Interestingly, M was the most conserved segment showing the lowest values in nucleotide and haplotype diversity. In particular, Tajima’s D provided negative values from −1.29 to −2.09 in all of the segments, indicating population size expansion or evidence for purifying selection, which is responsible for the preservation of the adaptive characteristics of organisms. Therefore, genetic variation within the main H3N2 CIVs was not dramatically dynamic, and adaptation of H3N2 CIVs was expected to be mediated by subtle changes.

**Table 3:** Summary statistics for molecular variation in eight segments

Population genetic indexes		PB2	PB1	PA	HA	NP	NA	M	NS
Polymorphism	Sequences	44	43	43	50	45	48	44	43
	*S* ^a^	55	46	50	154	125	103	50	74
	Pi^b^	31	23	27	66	75	63	20	38
	*h* ^c^	27	26	26	46	34	33	26	30
	Hd^d^	0.964	0.951	0.955	0.996	0.977	0.946	0.885	0.976
	*π* ^e^	0.011	0.011	0.010	0.011	0.010	0.009	0.008	0.013
	*κ* ^f^	7.77	7.01	5.82	14.71	15.05	12.31	6.01	8.47
Neutrality test	Tajima’s D^g^	−1.40	−1.29	−1.79	−2.09	−1.75	−1.72	−1.71	−1.84

^a^Number of polymorphic (segregating) sites

^b^Parsimony informative sites

^c^Number of haplotypes

^d^Haplotype diversity

^e^Nucleotide diversity

^f^Average number of nucleotide differences

^g^Tajima’s D to examine the demographic trends

### Median-joining network based on HA and NA

**Fig. 3:**
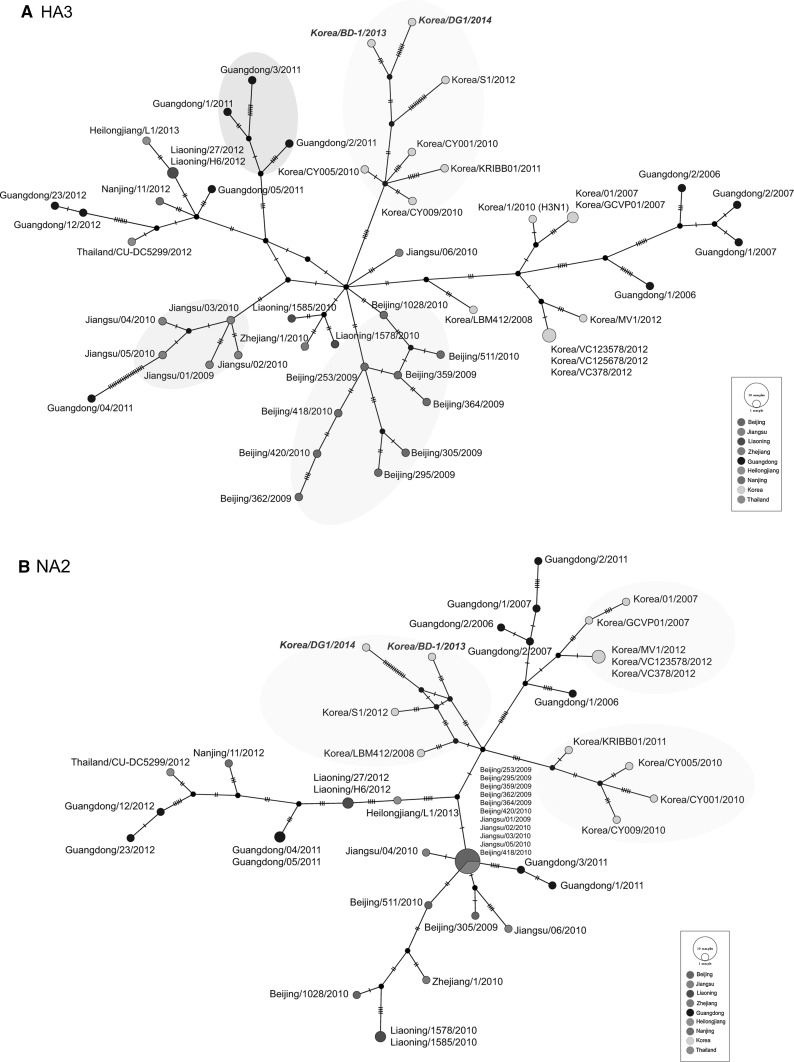
Median-joining phylogenetic network of H3N2 CIVs based on HA and NA. Each unique sequence is represented by a *circle*, the size of which is proportional to haplotype frequency. The *small lines* and *black dots* reflect mutations and median vectors, respectively. H3N2 CIVs are *colored* according to geographical locations

The estimated genealogical relationships of haplotypes from two antigenic proteins (HA and NA) are shown in Fig. 3. Largely, HA and NA showed sporadic and regional distribution patterns, according to geographical affinities and isolation years. For instance, in 46 HA haplotype sequences, two distinct Korean groups were identified: Guangdong (2006–2007) and Korean isolates (2010–2014). At the same time, Chinese H3N2 CIVs showed multiple routes based on complex reticular events: Beijing (2009–2010), Jiangsu (2009–2010), Guangdong (2011), and multiple regions (2011–2013). In 33 NA, one major dispersion center was identified, which consisted of CIVs from two closely located regions: Beijing (2009–2010) and Jiangsu (2009–2010). In particular, in South Korea, three distinct groups with different routes were observed. The first group was related to the early isolates of Chinese in 2006–2007, and the other two showed different evolutionary pathways with more recent Korean isolates. Similar to the HA distribution pattern, Guangdong CIVs were mostly dispersed with other regional CIVs. In addition, when closely examining the mutation steps, it was evident that the branches of Korea/DG1/2014 in NA and Guangdong/04/2011 in HA were unusually long with more mutations.

### AMOVA test between Korean and Chinese H3N2 CIVs

To estimate population differentiation between Korea and China including Thailand, AMOVA was conducted, and its results are described in the following. When considering the *Φ* value of AMOVA, close to 0 indicates no differentiation between the overall population and its subpopulations, whereas close to 1 indicates highly differentiated populations. First, little differentiation among populations was observed within the same geographical region: *Φ*_SC_ = 0.06955 in HA and −0.01539 in NA. There was, however, substantial differentiation among regions: *Φ*_CT_ = 0.19194 in HA and 0.24270 in NA. As a result, the differences among populations in different regions responsible for nearly all of the differences among populations produced significant values: *Φ*_ST_ = 0.24814 (*p* < 0.001) in HA and 0.23104 (*p* < 0.001) in NA. Thus, the results suggested that the independent evolution process is ongoing between the two countries.

### Amino acid changes

The amino acid residues of the eight segments of the two Korean isolates did not significantly differ from other H3N2 CIVs, based on the specific sites previously described in recent reports (data not shown). Only 11 amino acid changes were obviously observed as possible adaptation mutations for the two isolates (Table 4). For details, the NA segment showed more amino acid changes: NA stalk insertion was not recovered from the two Korean CIVs; three mutations in antigenic sites—B (R201K), C (S336N), and D (N467D)—were identified. In HA, one mutation (A10V) of the glycosylation site at 8–10 was observed, which overlapped with the H3N2 CIV host adaptation site. Other changes in H3N2 CIVs occurred in additional host adaptation sites of PB2 (M76I), PB1 (S216 N), PA (Y65H and C241Y), and NP (I109V). Interestingly, the unique mutation for the two isolates was discovered at 327 in PA (E- > K). In general, A/canine/Korea/BD-1/2013 had more mutations in the ribonucleoprotein (RNP) complex, consisting of PB2, PB1, PA, and NP, whereas A/canine/Korea/DG1/2014 harbored four mutations in antigenic sites of HA and NA.

**Table 4:** Mutations in the deduced amino acids of segments between newly isolated Korean CIVs and other IAVs from different hosts

Segments	PB2	PB1	PA			NP	HA1		NA			
Functions^a^	Host	Host	Host	Host	N/D	Host	Glycosylation	Host	Stalk insertion	antigenic sites (B)	antigenic sites (C)	antigenic sites (D)
Positions^b^	76	216	65	241	327	109	8–10	10	77–78	199–202	336	464–471
H3N2 human	T	S	L	C	E	V	NST	T	–	DDKN	S	ADINLMPI
H3N2 swine	T	S	S	C	E	I	NSM	M	–	DDKN	S	ADINLMPI
H3N2 avian	T	S	S	C	E	I	NNT	T	–	DDRN	S	ANINFMPI
H3N2 canine^c^	M	S	Y	C	E	I	NNA	A	KE	DDRN	S	ANINFMPI
Korea/BD-1/2013	**I**	**N**	**H**	**Y**	**K**	**V**	NNA	A	–	DD**K**N	S	ANINFMPI
Korea/DG1/2014	M	S	Y	C	**K**	**V**	NN**V**	**V**	–	DDRN	**N**	ANI**D**FMPI

Amino acid resides different from dominant H3N2 CIV type were denoted by bold letters

*N/D* not defined

^a^Associated host adaption sites (Host) were described in Zhu [11], Hu [60], Bunpapong [19], Su [66], Lin [20], and Taubenberger [61]

^b^Amino acid positions based on A/canine/Guandong/3/2011 genomic sequences (Supplementary Table S2)

^c^A majority of amino acid residues occurred in H3N2 CIVs

## Discussion

### CIV genotyping

In this study, the analyzed genomic genotype of H3N2 CIVs showed that it was occasionally changed due to reassortment events with a pandemic 2009 strain since 2009, but one genotype, (*K, G, E, 3B, F, 2D, F, 1E*), is predominantly circulating in Asian regions. Notably, the general genotype pattern of domestic poultry (*K/G, G, D/E, 4, F, 6, F, 1E/1F/2A*) previously described in Dong et al. (2011) [45], is highly similar to the current genotype of H3N2 CIVs, indicating a close relationship with the origin of H3N2 CIVs. In particular, the genotypes of H3N2 AIVs from Korean domestic poultry, such as A/duck/Korea/U14-1/2007 (*K, G, D, 3B, F, 2D, F, 1E*) and A/duck/Korea/U4-1/2007 (*G, G, E, 3B, F, 2D, F, 1E*), determined in FluGenome, were almost same as the H3N2 CIVs.

Using FluGenome, 10 % delimitation by p-distance to classify IAVs lineages seemed inappropriate for the current CIVs because the criterion could not fully classify the lineages caused by the recent interspecies transmission due to minor differences (Fig. 1; A/canine/Korea/MV1/2012 in M and A/canine/Guangdong/2/2007 in NS). Therefore, it was believed that its classification should be more adjusted to distinguish new lineages from recent years.

### Phylogenetic analysis and median-joining network

Phylogenetic analyses for each segment showed that lineages caused by reassortment events were clearly separated from the H3N2 CIVs with different genotypes, but depending on historical origins, the reassortant lineages showed a phylogenetic affinity with the H3N2 CIVs. In this study, the full genomic segments of two Korean strains revealed that they are closely related to a group consisting of the most recent Korean H3N2 CIVs isolated from 2010 to 2012 (Fig. 2).

In addition, median-joining networking, based on the HA and NA of H3N2 CIVs, illustrated several interesting relationships among them, which were presumably undescribed (Fig. 3). First, since the early detection (2007–2008) of H3N2 CIVs, Korean and Chinese CIVs have appeared to evolve into distinct groups, indicating that the movement of the dog population between the two countries was well controlled during recent years; otherwise, migratory birds intermediating H3N2 CIVs might have influenced the limited distribution. As shown in Fig. 3, in South Korea, two distinct groups in HA and three in NA were identified, which were distinct from the Chinese main group. Next, intrasubtypic reassortants have occurred the most often in Guangdong in China since the first isolation (2006) in both HA and NA. For this reason, the haplotype of H3N2 CIVs from the Guangdong was sporadically observed across locations within China, suggesting that H3N2 CIVs might have derived from frequent local travel and animal trade. Indeed, Guangdong province, which is located very close to Hong Kong, where frequent animal trade occurs, was expected to be a risky location for H3N2 CIV infections and their spread [16].

### Genetic variation of each segment

According to the population genetic indices, each segment of H3N2 CIVs was more affected by purifying selection; thus, mutations occurred and accumulated at silent sites, showing that nucleotide diversity is relatively small but with many segregating sites (Table 3). In the comparison of the deduced amino acids based on specific functional sites between the newly isolated Korean CIVs and other H3N2 CIVs, the total of 11 changes was observed (Table 4). The mutations were mostly observed in antigenic sites of NA segments, indicating that these changes influenced on the host adaptation process. Further, the remaining mutations of host-specific residues were associated with specific binding domains of RNP complex (PB2 [M76I], PB1 [S216N], PA [Y65H], and NP [I109 V]), which were previously described in Zhu et al. [11], Chen et al. [58], Naffakh et al. [59], and Hu et al. [60]. The mutations were interesting that the RNP complex is involved in replication and interacts with host factors; thus, it was expected to have host specificity. In addition, the independent emergence of the PA E327 K mutation for the two isolates was first discovered among H3N2 CIVs. Finally, one unique change was observed in the nuclear localization signal of PA(C241Y), which was previously mentioned as 1918 H1N1-specific marker [61]. Based on the comparison with amino acids of human, swine, and avian hosts, a few changes of the two H3N2 CIVs were relatively unique in Table 4. However, if considering its frequency in currently available influenza A viral sequences, the mutations were possible events. Thus, the biological processes caused by those changes need to be further examined by functional studies in vivo.

## Conclusions

In this study, for an explicit and rapid approach to identifying each segment lineage of H3N2 CIVs, genotype classification suggested by FluGenome was attempted for the first time. For the approaches, comprehensive molecular analyses were conducted to reveal the evolutionary history of 57 CIVs. In addition, two newly isolated Korean CIVs, A/canine/Korea/BD-1/2013 and A/canine/Korea/DG1/2014, were characterized using complete genomic sequences. Phylogenetic relationships among the CIVs based on genotype profiling and genetic analysis for each segment within the H3N2 CIVs showed that the two Korean CIVs were closely related to the recent Korean isolates (2010–2012), which are predominantly circulating in Asia with genotype patterns *K, G, E, 3B, F, 2D, F,* and *1E*. Analysis of the amino acids of two H3N2 CIVs showed 11 mutations distinct from general H3N2 CIVs occurring in the binding domains of ribonucleoprotein (RNP) complex, antigenic sites of NA, and a glycosylation site of HA. In addition, median-joining networking, based on HA and NA, showed diversification within the H3N2 CIVs processed independently between Korea and China since the first outbreak in 2006–2007. In general, H3N2 CIVs tended to cluster together according to geographic affinities, but intrasubtypic reassortment events were often observed in Guandong province in China, indicating the possibility of frequent animal trade.

## Electronic supplementary material

Supplementary material 1 (PDF 118 kb)

Supplementary material 2 (DOC 121 kb)

Supplementary material 3 (DOCX 12 kb)
