# Supplementary figures and images for: Molecular analyses of H3N2 canine influenza viruses isolated from Korea during 2013–2014
Source: Virus Genes. 2016 Jan 25;52:204–17. doi: 10.1007/s11262-015-1274-x (PMC4792367; doi:10.1007/s11262-015-1274-x)

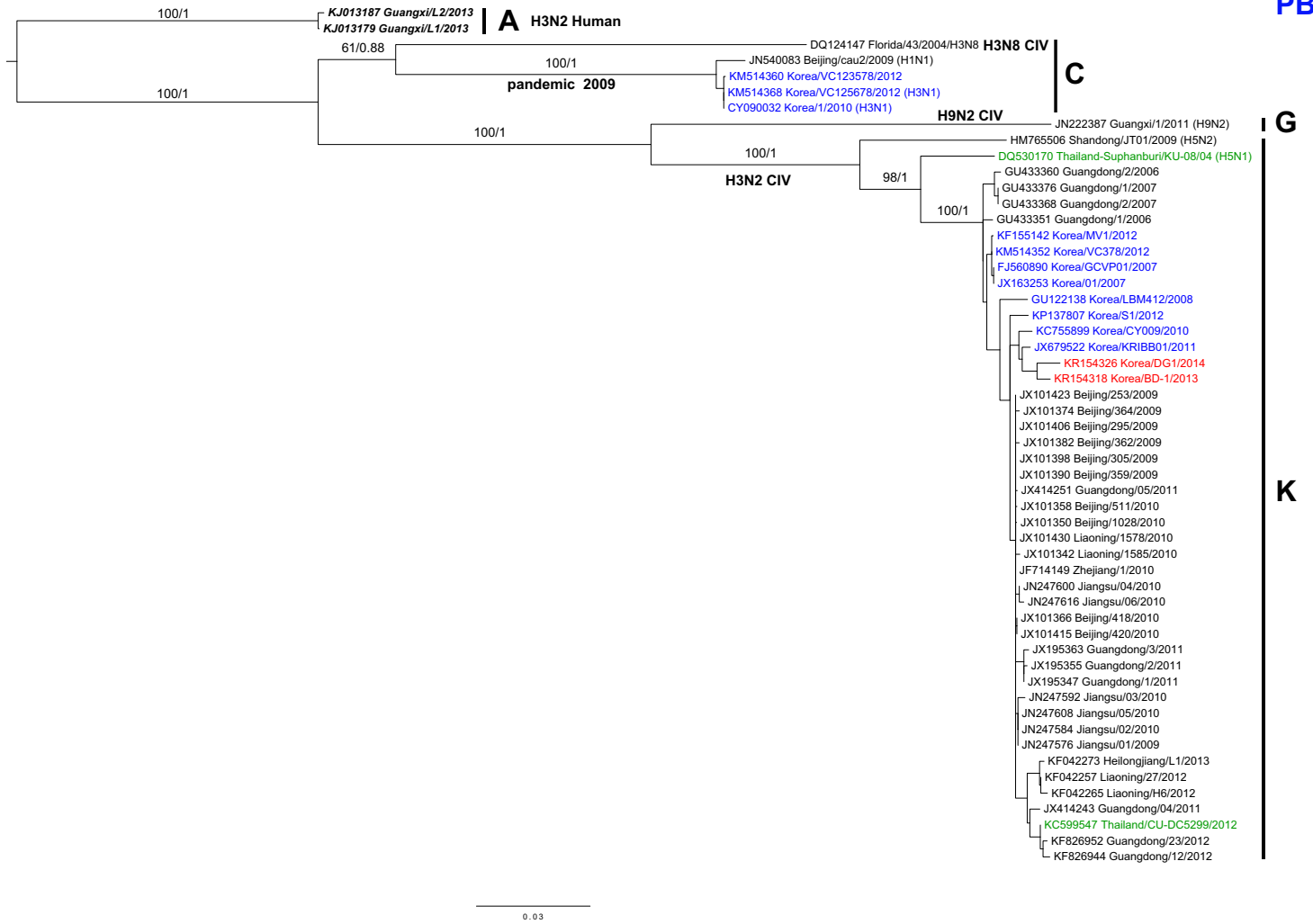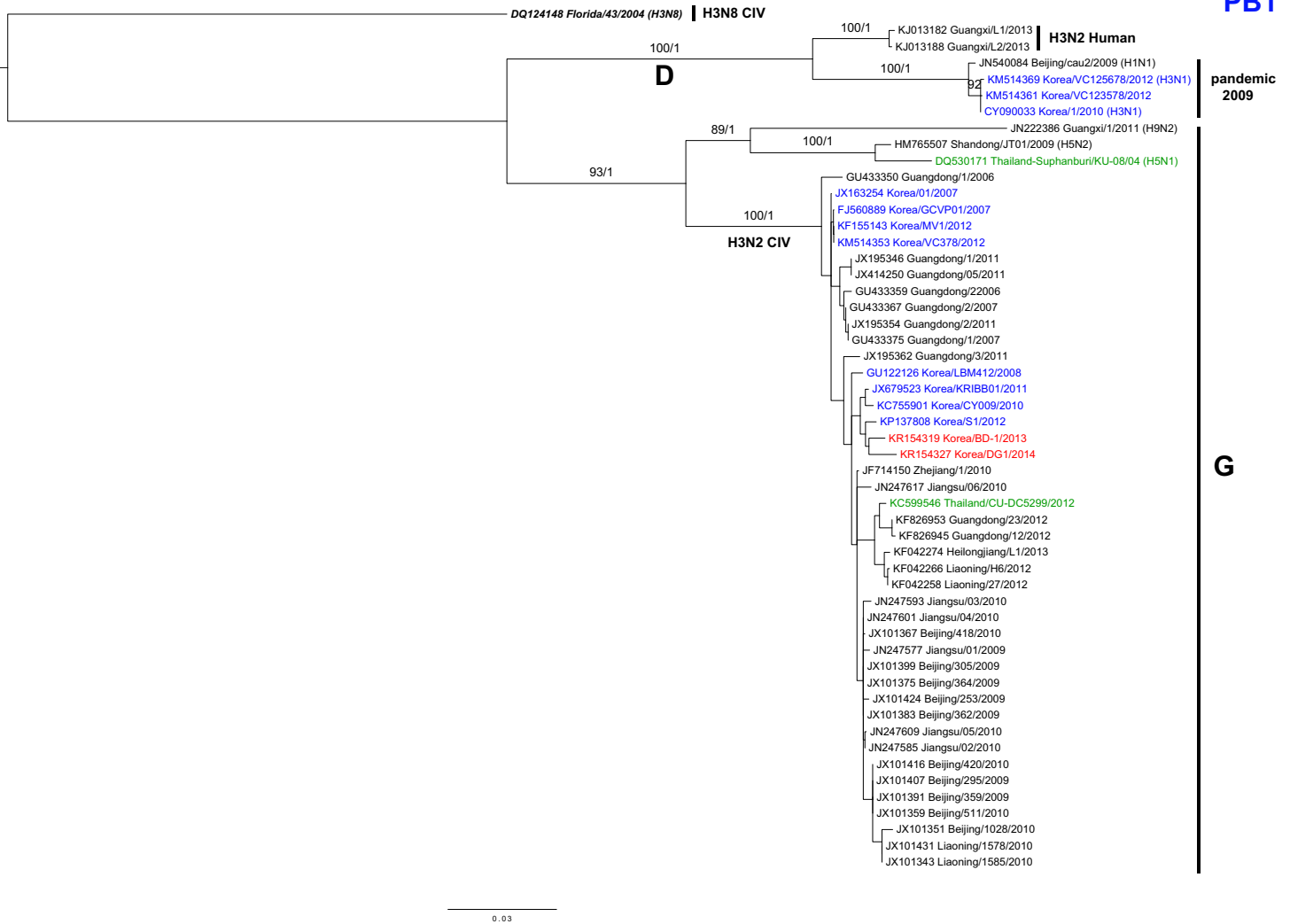

PA

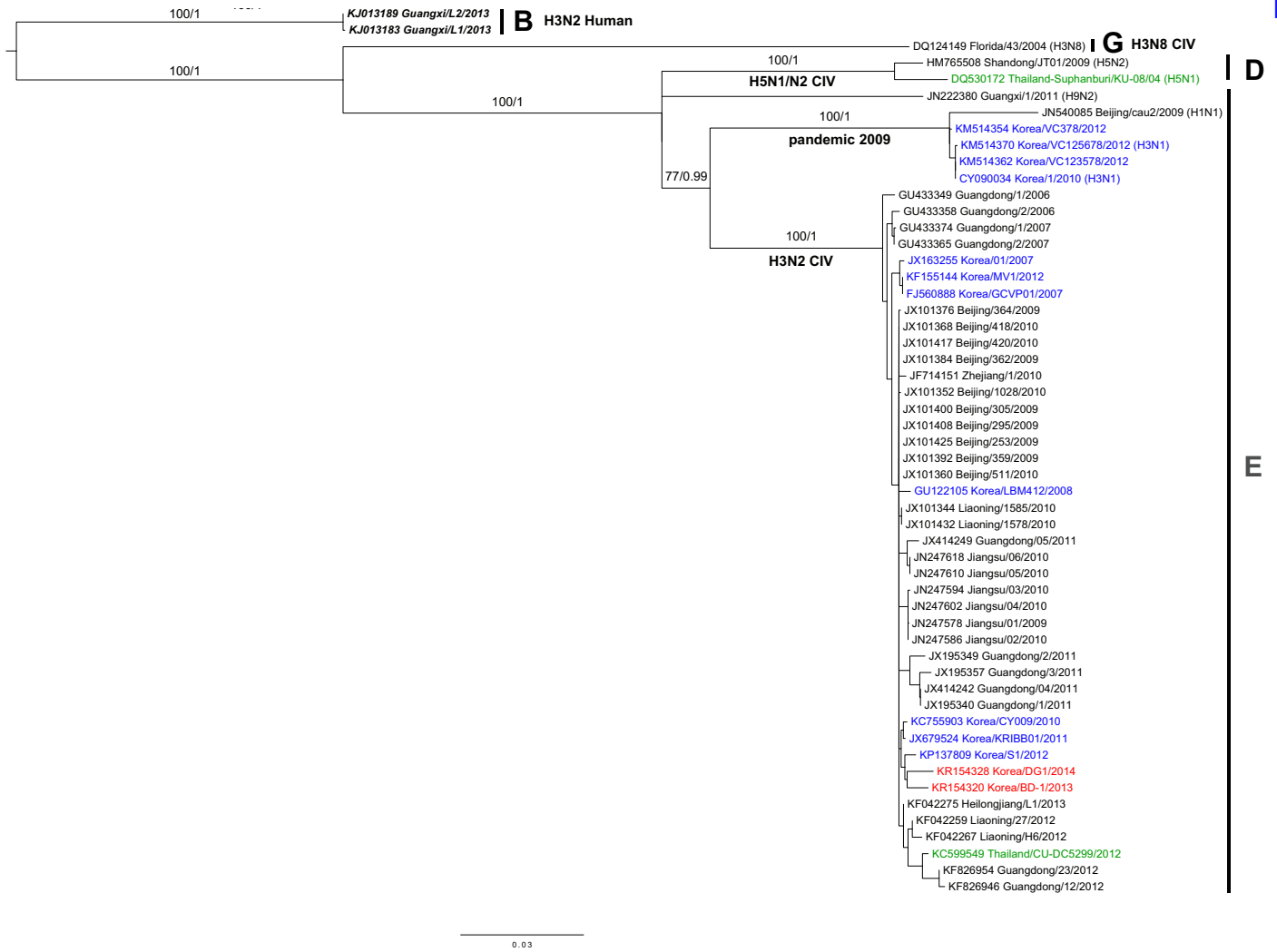

E

NP

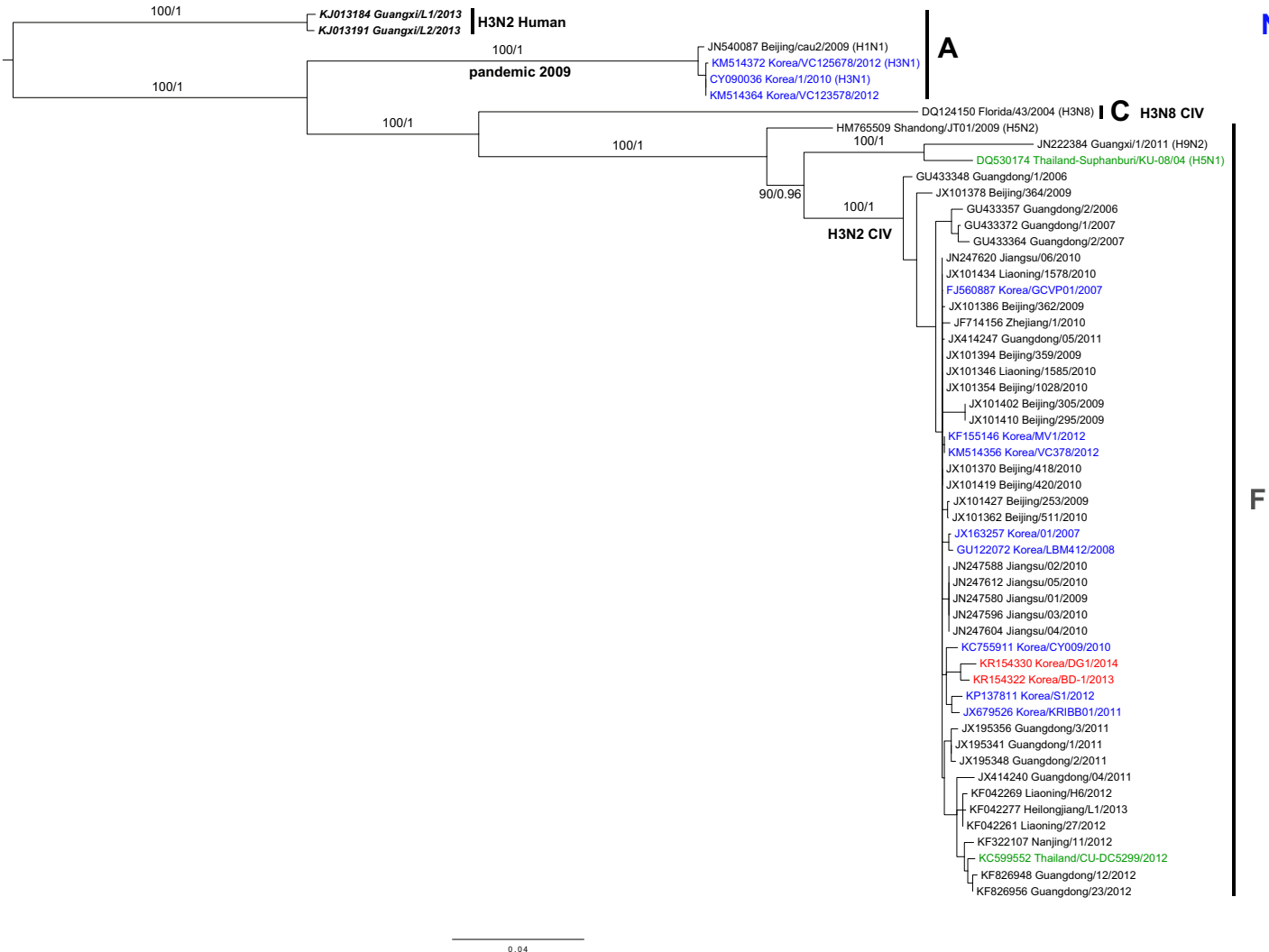

F

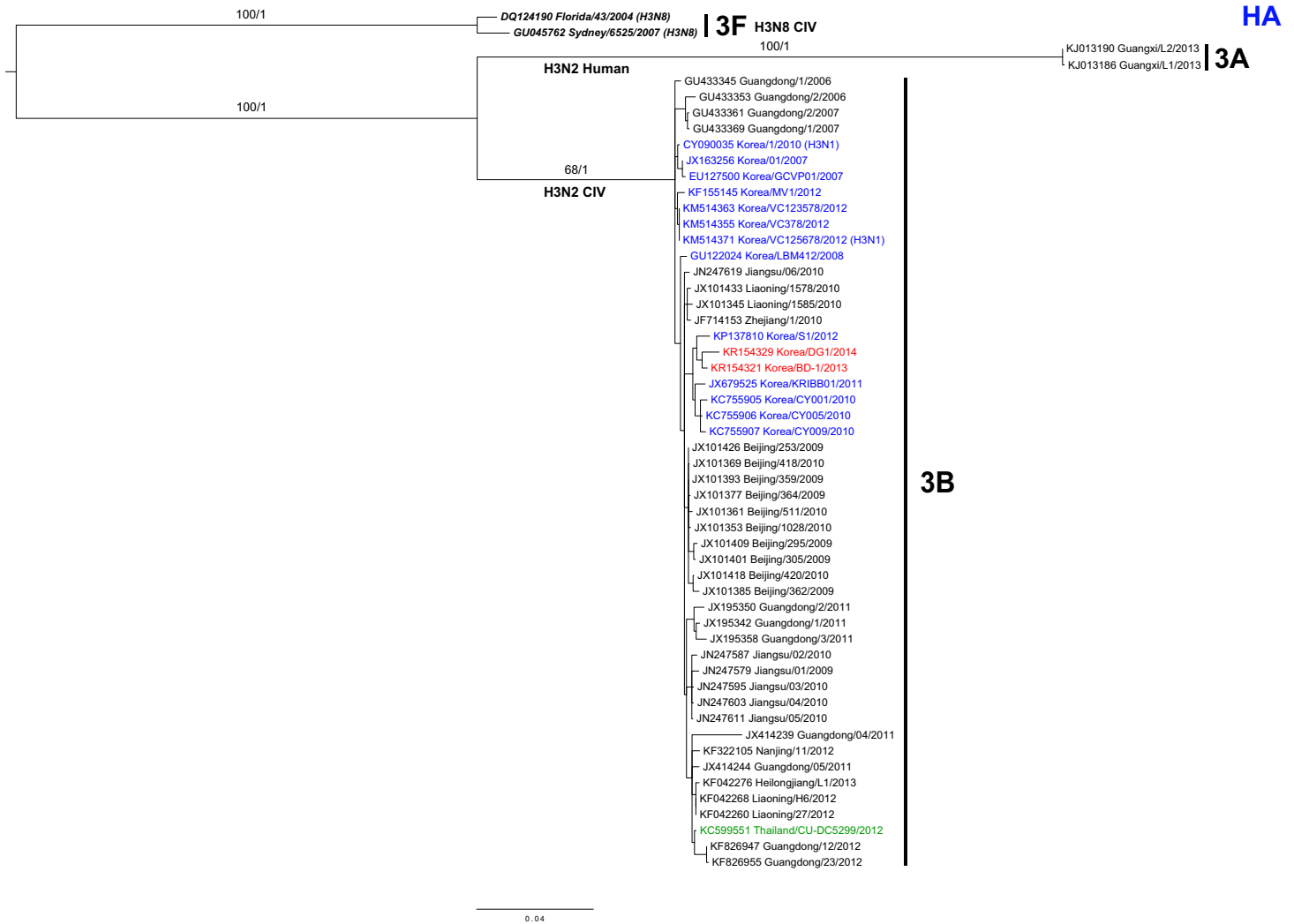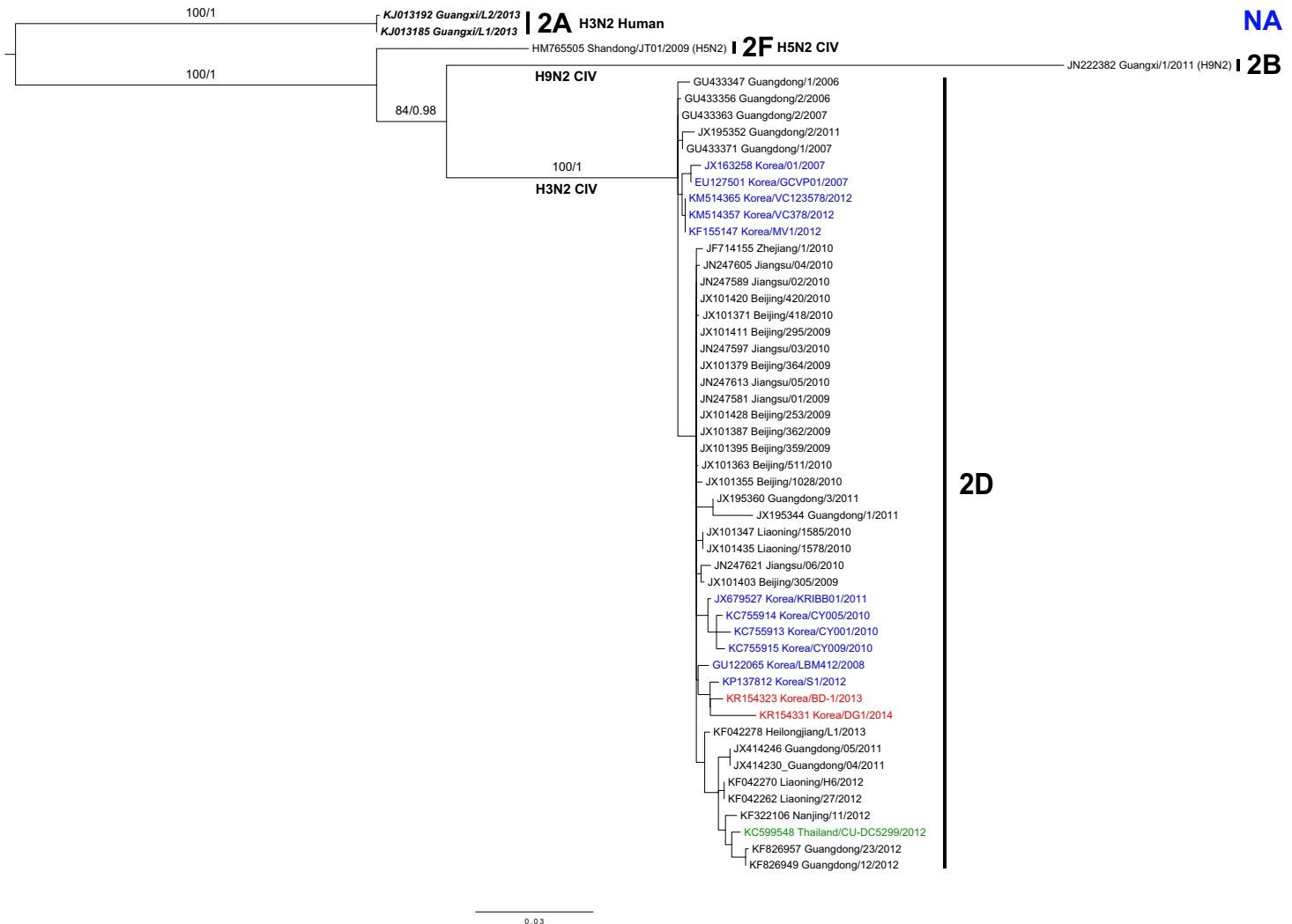

Supplement: Supplementary file 1 — Supplementary material 1 (PDF 118 kb) [file 11262_2015_1274_MOESM1_ESM.pdf]
