# Supplementary material for: Molecular analyses of H3N2 canine influenza viruses isolated from Korea during 2013–2014
Source: Virus Genes. 2016 Jan 25;52:204–17. doi: 10.1007/s11262-015-1274-x (PMC4792367; doi:10.1007/s11262-015-1274-x)
Supplement: Supplementary file 2 — Supplementary material 2 (DOC 121 kb) [file 11262_2015_1274_MOESM2_ESM.doc]

**S1. Table. 59 CIV genomic sequence data analyzed in this study**

| No | CIVs & Subtypes |  | PB2 | PB1 | PA | HA | NP | NA | M | NS |
| --- | --- | --- | --- | --- | --- | --- | --- | --- | --- | --- |
| 1 | A/canine/Korea/01/2007 | H3N2 | JX163253 | JX163254 | JX163255 | JX163256 | JX163257 | JX163258 | JX163259 | JX163260 |
| 2 | A/Canine/Korea/GCVP01/2007 | H3N2 | FJ560890 | FJ560889 | FJ560888 | EU127500 | FJ560887 | EU127501 | FJ560885 | FJ560886 |
| 3 | A/canine/Korea/LBM412/2008 | H3N2 | GU122138 | GU122126 | GU122105 | GU122024 | GU122072 | GU122065 | GU122045 | GU122091 |
| 4 | A/canine/Korea/SNU9046/2009 | H3N2 |  |  |  |  |  |  |  | JX273029 |
| 5 | A/canine/Korea/CY001/2010 | H3N2 |  |  |  | KC755905 |  | KC755913 | KC755919 |  |
| 6 | A/canine/Korea/CY005/2010 | H3N2 |  |  |  | KC755906 |  | KC755914 | KC755920 |  |
| 7 | A/canine/Korea/CY009/2010 | H3N2 | KC755899 | KC755901 | KC755903 | KC755907 | KC755911 | KC755915 | KC755921 | KC755925 |
| 8 | A/canine/Korea/1/2010 | H3N1 | CY090032 | CY090033 | CY090034 | CY090035 | CY090036 | 양식의 맨 위  CY090037 | CY090038 | CY090039 |
| 9 | A/canine/Korea/KRIBB01/2011 | H3N2 | JX679522 | JX679523 | JX679524 | JX679525 | JX679526 | JX679527 | JX679528 | JX679529 |
| 10 | A/canine/Korea/MV1/2012 | H3N2 | KF155142 | KF155143 | KF155144 | KF155145 | KF155146 | KF155147 | KF155148 | KF155149 |
| 11 | A/canine/Korea/S1/2012 | H3N2 | KP137807 | KP137808 | KP137809 | KP137810 | KP137811 | KP137812 | KP137813 | KP137814 |
| 12 | A/canine/Korea/VC378/2012 | H3N2 | KM514352 | KM514353 | KM514354 | KM514355 | KM514356 | KM514357 | KM514358 | KM514359 |
| 13 | A/canine/Korea/VC123578/2012 | H3N2 | KM514360 | KM514361 | KM514362 | KM514363 | KM514364 | KM514365 | KM514366 | KM514367 |
| 14 | A/canine/Korea/VC125678/2012 | H3N1 | KM514368 | KM514369 | KM514370 | KM514371 | KM514372 | KM514373 | KM514374 | KM514375 |
| 15 | ***A/canine/Korea/BD-1/2013 **** | H3N2 | KR154318 | KR154319 | KR154320 | KR154321 | KR154322 | KR154323 | KR154324 | KR154325 |
| 16 | ***A/canine/Korea/DG1/2014 **** | H3N2 | KR154326 | KR154327 | KR154328 | KR154329 | KR154330 | KR154331 | KR154332 | KR154333 |
| 17 | A/canine/Guangdong/1/2006 | H3N2 | GU433351 | GU433350 | GU433349 | GU433345 | GU433348 | GU433347 | GU433346 | GU433352 |
| 18 | A/canine/Guangdong/2/2006 | H3N2 | GU433360 | GU433359 | GU433358 | GU433353 | GU433357 | GU433356 | GU433355 | GU433354 |
| 19 | A/canine/Guangdong/1/2007 | H3N2 | GU433376 | GU433375 | GU433374 | GU433369 | GU433372 | GU433371 | GU433370 | GU433373 |
| 20 | A/canine/Guangdong/2/2007 | H3N2 | GU433368 | GU433367 | GU433365 | GU433361 | GU433364 | GU433363 | GU433362 | GU433366 |
| 21 | A/canine/Guangdong/1/2011 | H3N2 | JX195347 | JX195346 | JX195340 | JX195342 | JX195341 | JX195344 | JX195343 | JX195345 |
| 22 | A/canine/Guangdong/05/2011 | H3N2 | JX414251 | JX414250 | JX414249 | JX414244 | JX414247 | JX414246 | JX414245 | JX414248 |
| 23 | A/canine/Guangdong/2/2011 | H3N2 | JX195355 | JX195354 | JX195349 | JX195350 | JX195348 | JX195352 | JX195351 | JX195353 |
| 24 | A/canine/Guangdong/3/2011 | H3N2 | JX195363 | JX195362 | JX195357 | JX195358 | JX195356 | JX195360 | JX195359 | JX195361 |
| 25 | A/canine/Guangdong/04/2011 | H3N2 | JX414243 |  | JX414242 | JX414239 | JX414240 | JX414230 | JX414229 | JX414241 |
| 26 | A/canine/Guangdong/12/2012 | H3N2 | KF826944 | KF826945 | KF826946 | KF826947 | KF826948 | KF826949 | KF826950 | KF826951 |
| 27 | A/canine/Guangdong/23/2012 | H3N2 | KF826952 | KF826953 | KF826954 | KF826955 | KF826956 | KF826957 | KF826958 | KF826959 |
| 28 | A/canine/Beijing/cau2/2009 | H1N1 | JN540083 | JN540084 | JN540085 | JN540086 | JN540087 | JN540088 | JN540089 | JN540090 |
| 29 | A/canine/Beijing/253/2009 | H3N2 | JX101423 | JX101424 | JX101425 | JX101426 | JX101427 | JX101428 | JX101422 | JX101429 |
| 30 | A/canine/Beijing/295/2009 | H3N2 | JX101406 | JX101407 | JX101408 | JX101409 | JX101410 | JX101411 | JX101412 | JX101413 |
| 31 | A/canine/Beijing/305/2009 | H3N2 | JX101398 | JX101399 | JX101400 | JX101401 | JX101402 | JX101403 | JX101404 | JX101405 |
| 32 | A/canine/Beijing/359/2009 | H3N2 | JX101390 | JX101391 | JX101392 | JX101393 | JX101394 | JX101395 | JX101396 | JX101397 |
| 33 | A/canine/Beijing/362/2009 | H3N2 | JX101382 | JX101383 | JX101384 | JX101385 | JX101386 | JX101387 | JX101388 | JX101389 |
| 34 | A/canine/Beijing/364/2009 | H3N2 | JX101374 | JX101375 | JX101376 | JX101377 | JX101378 | JX101379 | JX101380 | JX101381 |
| 35 | A/canine/Beijing/418/2010 | H3N2 | JX101366 | JX101367 | JX101368 | JX101369 | JX101370 | JX101371 | JX101372 | JX101373 |
| 36 | A/canine/Beijing/420/2010 | H3N2 | JX101415 | JX101416 | JX101417 | JX101418 | JX101419 | JX101420 | JX101414 | JX101421 |
| 37 | A/canine/Beijing/511/2010 | H3N2 | JX101358 | JX101359 | JX101360 | JX101361 | JX101362 | JX101363 | JX101365 | JX101364 |
| 38 | A/canine/Beijing/1028/2010 | H3N2 | JX101350 | JX101351 | JX101352 | JX101353 | JX101354 | JX101355 | JX101356 | JX101357 |
| 39 | A/canine/Liaoning/1578/2010 | H3N2 | JX101430 | JX101431 | JX101432 | JX101433 | JX101434 | JX101435 | JX101436 | JX101437 |
| 40 | A/canine/Liaoning/1585/2010 | H3N2 | JX101342 | JX101343 | JX101344 | JX101345 | JX101346 | JX101347 | JX101348 | JX101349 |
| 41 | A/canine/Liaoning/27/2012 | H3N2 | KF042257 | KF042258 | KF042259 | KF042260 | KF042261 | KF042262 | KF042263 | KF042264 |
| 42 | A/canine/Liaoning/H6/2012 | H3N2 | KF042265 | KF042266 | KF042267 | KF042268 | KF042269 | KF042270 | KF042271 | KF042272 |
| 43 | A/canine/Jiangsu/01/2009 | H3N2 | JN247576 | JN247577 | JN247578 | JN247579 | JN247580 | JN247581 | JN247582 | JN247583 |
| 44 | A/canine/Jiangsu/02/2010 | H3N2 | JN247584 | JN247585 | JN247586 | JN247587 | JN247588 | JN247589 | JN247590 | JN247591 |
| 45 | A/canine/Jiangsu/03/2010 | H3N2 | JN247592 | JN247593 | JN247594 | JN247595 | JN247596 | JN247597 | JN247598 | JN247599 |
| 46 | A/canine/Jiangsu/04/2010 | H3N2 | JN247600 | JN247601 | JN247602 | JN247603 | JN247604 | JN247605 | JN247606 | JN247607 |
| 47 | A/canine/Jiangsu/05/2010 | H3N2 | JN247608 | JN247609 | JN247610 | JN247611 | JN247612 | JN247613 | JN247614 | JN247615 |
| 48 | A/canine/Jiangsu/06/2010 | H3N2 | JN247616 | JN247617 | JN247618 | JN247619 | JN247620 | JN247621 | JN247622 | JN247623 |
| 49 | A/canine/Zhejiang/1/2010 | H3N2 | JF714149 | JF714150 | JF714151 | JF714153 | JF714156 | JF714155 | JF714154 | JF714152 |
| 50 | A/canine/Nanjing/11/2012 | H3N2 |  |  |  | KF322105 | KF322107 | KF322106 |  |  |
| 51 | A/canine/Heilongjiang/L1/2013 | H3N2 | KF042273 | KF042274 | KF042275 | KF042276 | KF042277 | KF042278 | KF042279 | KF042280 |
| 52 | A/canine/Guangxi/1/2011 | H9N2 | JN222387 | JN222386 | JN222380 | JN222381 | JN222384 | JN222382 | JN222383 | JN222385 |
| 53 | A/canine/Guangxi/L1/2013 | H3N2 | KJ013179 | KJ013182 | KJ013183 | KJ013186 | KJ013184 | KJ013185 | KJ013180 | KJ013181 |
| 54 | A/canine/Guangxi/L2/2013 | H3N2 | KJ013187 | KJ013188 | KJ013189 | KJ013190 | KJ013191 | KJ013192 | KJ013193 | KJ013194 |
| 55 | A/dog/Shandong/JT01/2009 | H5N2 | HM765506 | HM765507 | HM765508 | HM765504 | HM765509 | HM765505 | HM765510 | HM765511 |
| 56 | A/canine/Thailand/CU-DC5299/2012 | H3N2 | KC599547 | KC599546 | KC599549 | KC599551 | KC599552 | KC599548 | KC599545 | KC599550 |
| 57 | A/dog/Thailand-Suphanburi/KU-08/04 | H5N1 | DQ530170 | DQ530171 | DQ530172 | DQ530173 | DQ530174 | DQ530175 | DQ530176 | DQ530177 |
| 58 | A/canine/Florida/43/2004 | H3N8 | DQ124147 | DQ124148 | DQ124149 | DQ124190 | DQ124150 | DQ124151 | DQ124152 | DQ124153 |
| 59 | A/canine/Sydney/6525/2007 | H3N8 |  |  |  | GU045762 |  | 양식의 맨 위  GU045765 | 양식의 맨 위  GU045768 |  |

**S2. Table. Estimates of evolutionary divergence over sequence pairs between groups in each segment: (a) PB1 and PB2; (b) NP and PA; (c) HA3 and NA2**

(a) PB1 and PB2

|  | H3N2 CIV | H5N2 | H5N1 | H9N2 | H3N2 Human | pandemic 2009 | H3N8 CIV |
| --- | --- | --- | --- | --- | --- | --- | --- |
| H3N2 CIV |  | 0.080 | **0.047^a^** | 0.139 | **0.176^b^** | 0.164 | 0.175 |
| H5N2 | **0.088^a^** |  | 0.065 | 0.134 | 0.176 | 0.161 | 0.162 |
| H5N1 | 0.095 | 0.020 |  | 0.135 | 0.168 | 0.156 | 0.167 |
| H9N2 | 0.106 | 0.093 | 0.095 |  | 0.179 | 0.165 | 0.165 |
| H3N2 Human | 0.135 | 0.139 | 0.138 | 0.148 |  | 0.160 | 0.161 |
| pandemic 2009 | 0.143 | 0.150 | 0.150 | 0.161 | 0.058 |  | 0.135 |
| H3N8 CIV | **0.187^b^** | 0.188 | 0.183 | 0.179 | 0.181 | 0.185 |  |

Lower matrix: PB1 segment; Upper matrix: PB2 segment.

^a^ Minimum divergence and ^b^ maximum divergence from H3N2 CIVs is represented by bold numbers for each segment.

(b) NP and PA

|  | H3N2 CIV | H5N2 | H9N2 | H5N1 | H3N8 CIV | pandemic 2009 | H3N2 Human |
| --- | --- | --- | --- | --- | --- | --- | --- |
| H3N2 CIV |  | 0.106 | **0.100^a^** | 0.104 | 0.170 | 0.107 | **0.184^b^** |
| H5N2 | **0.063^a^** |  | 0.107 | 0.020 | 0.163 | 0.120 | 0.169 |
| H9N2 | 0.086 | 0.086 |  | 0.111 | 0.158 | 0.116 | 0.171 |
| H5N1 | 0.080 | 0.068 | 0.044 |  | 0.163 | 0.117 | 0.167 |
| H3N8 CIV | 0.161 | 0.152 | 0.167 | 0.167 |  | 0.175 | 0.173 |
| pandemic 2009 | 0.178 | 0.166 | 0.182 | 0.172 | 0.168 |  | 0.179 |
| H3N2 Human | **0.191^b^** | 0.189 | 0.191 | 0.187 | 0.191 | 0.180 |  |

Lower matrix: NP segment; Upper matrix: PA segment.

(c) HA3 and NA2

|  | H3N2 CIV | H5N2 | H9N2 | H3N2 Human | H3N8 CIV |
| --- | --- | --- | --- | --- | --- |
| H3N2 CIV |  | **0.101^a^** | 0.162 | **0.182^b^** | na |
| H5N2 | na |  | 0.157 | 0.163 | na |
| H9N2 | na | na |  | 0.204 | na |
| H3N2 Human | **0.183^a^** | na | na |  | na |
| H3N8 CIV | **0.221^b^** | na | na | 0.251 |  |

Lower matrix: HA segment; Upper matrix: NA segment; ‘na’ means ‘not available’ for the analysis
