# Supplementary material for: Molecular analyses of H3N2 canine influenza viruses isolated from Korea during 2013–2014
Source: Virus Genes. 2016 Jan 25;52:204–17. doi: 10.1007/s11262-015-1274-x (PMC4792367; doi:10.1007/s11262-015-1274-x)
Supplement: Supplementary file 3 — Supplementary material 3 (DOCX 12 kb) [file 11262_2015_1274_MOESM3_ESM.docx]

**Supporting Information**

**S1. Fig. Phylogenetic trees of CIVs according to genotypes based on PB1, PB2, NP, PA, HA and NA.**Korean isolates are shown in blue; CIVs isolated in the present study are in red, and the Thailand isolate is in green. The remaining isolates are from China. Numbers at nodes indicate support values for maximum likelihood (RAxML) and posterior probabilities for Bayesian inference (MrBayes). Only support values greater than 50 are shown. Genotypes for each group are indicated with vertical lines. Each tree is rooted on the most distant group or isolate, based on p-genetic distance.

**S1.Table. 59 CIV genomic sequence data analyzed in this study.**

**S2. Table. Estimates of evolutionary divergence over sequence pairs between groups in each segment: PB1 and PB2; NP and PA; HA3 and NA2.**

**S3. Phylogenetic analysis descriptions**

RAxML and MrBayes analyses were conducted for each segment under the GTR model, with G (a gamma distribution of among-site rate variation) as the closest model suggested by jModelTest. For the genome-wide phylogenetic analysis, three different phylogenetic methods – RAxML, Garli, and MrBayes – were used for the topology comparison under the best-fit partitioning schemes suggested by PartitionFinder.

For the two ML analysis, bootstrap supports were calculated using 1,000 bootstrap replicates for RAxML and 100 for Garli. The bootstrap tree was summarized with 50% majority rule consensus trees by the SumTrees script (version 3.3.1) implemented in DendroPy, version 3.12.0 [53]. In the Bayesian analysis, two runs with four chains were run for 10 million generations, and trees were sampled every 1000 generations. The following criteria were used to evaluate convergence: (a) the average standard deviation of split frequencies < 0.01; (b) potential scale reduction factor (PSRF) close to 1; and (c) an effective sample size (ESS) >200 for the posterior probabilities and parameters evaluated in Tracer, version 1.6[54]. The first 25% of tree samples prior to reaching a stationary posterior distribution were discarded as the burin-in, and the remaining trees were used to generate a majority rule consensus tree.
